# Epidemiology of diabetes mellitus, pre-diabetes, undiagnosed and uncontrolled diabetes in Central Iran: results from Yazd health study

**DOI:** 10.1186/s12889-020-8267-y

**Published:** 2020-02-03

**Authors:** Masoud Mirzaei, Masoud Rahmaninan, Mohsen Mirzaei, Azadeh Nadjarzadeh, Abbas Ali Dehghani tafti

**Affiliations:** 1 Yazd Cardiovascular Research Center, Shahid Sadoughi University of Medical Sciences, Jomhuri Blvd., Afshar Hospital, Yazd, Iran; 2 Yazd Diabetes Research Centre, Shahid Sadoughi University of Medical Sciences, Yazd, Iran; 3 Nutrition and Food Security Research Center, Department of Nutrition, School of Public Health, Shahid Sadoughi University of Medical Sciences, Yazd, Iran; 4 Department of Health Education and Promotion, Shahid Sadoughi University of Medical Sciences, Yazd, Iran

**Keywords:** Diabetes, Prevalence, Risk factors, Socio-economic, Iran

## Abstract

**Background:**

Over the past few decades, the prevalence of Diabetes Mellitus (DM) has risen rapidly in Iran and other low and middle-income countries. We investigated the prevalence of DM, pre-diabetes, undiagnosed and uncontrolled diabetes and its relationship with some associated socioeconomic factors in the Yazd Greater Area in Iran.

**Methods:**

Yazd Health Study is a longitudinal study conducted to determine the prevalence of non-communicable disease and related risk factors. In a two-step cluster sampling, 10,000 adults aged 20–69 years (200 clusters) were selected. In the recruitment phase, DM was considered if the patients had been either diagnosed DM by a physician or *had fasting blood glucose* ≥ 126 mg/dL. Chi square test was used for categorical variables to evaluate the differences and logistic regression model was applied to determine the predictors of diabetes.. *P*-value *<* 0.05 considered statistically significant.

**Results:**

Of the 9965 individuals recruited, the crude self-reported prevalence of DM was 14.1% (95% CI: 13.4–14.7). The prevalence was higher in women than men (15.6 vs.12.4%), significantly. The age-standardized prevalence of DM was 8%. The prevalence was 14.9% in Yazd local people and 8.6% in those residents migrated from other provinces (*P < 0.0001*). We showed a significant association between DM prevalence and age, education, marital status, unemployment, insurance status, and positive family history (*P < 0.0001*). The prevalence of DM diagnosed by phycisians was 16.1% in participants (age-standardized prevalence: 8.3%). The subset analysis showed that 4.8% of patients were not aware of their disease. The prevalence of pre-diabetes was 25.8%. Of those with diabetes, 58.3% were not adequately controlled, which is not statistically significant with socio-economic status.

**Conclusion:**

The current study showed a high prevalence of DM in Yazd Greater Area which is closely related to some socio-demographic factors. The high prevalence of pre-diabetes is alarming. Effective strategies for DM prevention should be introduced. The majority of people with diabetes are aware, but half of them are not controlled. The ineffective care plan currently in use, should be reviewed. Patients needs to be encouraged to improve their lifestyle. Active follow-up of patients is recommended to ensure continuity of care.

## Background

Diabetes mellitus (DM) is a major public health problem that is determined with impaired carbohydrate metabolism, protein, and fat due to unstable insulin secretion, insulin resistance secretion, or both [1]. With an 8.5% global prevalence of diabetes in 2014; various estimates suggest that the number of affected people will be risen from 422 million to 642 million in the world by 2040 [2, 3].

DM and its complications are among the most important causes of mortality.

Between 1990 and 2010; the rank of the disease has moved from 15 to 9, which corresponds to a 92.7% increase in the burden during the period [4]. Over the past decade, the prevalence of diabetes has risen rapidly due to an increase in the average age of the community, hereditary background, unhealthy dietary habits, sedentary lifestyle and increased obesity in line with the growth of urbanization [5, 6].

The prevalence of diabetes is estimated to be 8.5% in adults aged over 18 years in 2014 which has increased significantly over the past three decades, especially in low and middle-income countries [2]. In the Eastern Mediterranean Region (EMRO), the average prevalence of diabetes in adult population was 13.7% in 2014, which is the highest prevalence compared to other WHO regions [2].

In Iran, the prevalence of diabetes in adults aged 25–70 years was reported 11.9% (2011) which shows an increase of 35% compared to 2005. It is estimated that in the year 2030 nearly 9.2 million Iranians likely to have diabetes [7]. Many people with diabetes are unaware of their complications due to uncontrolled blood glucose level [8]. A significant percentage of patients are unaware of their illness (from 30% in Iran to 86% in Tunisia in the Middle East and from 24.1 to 75.1% in other parts of the world) [9, 10]. Delay in the diagnosis of DM increases the cost of management and reduces the prognosis of the disease [11].

Yazd, a world heritage city located in the center of Iran, has one of the highest recorded prevalence of DM in Iran [12]. The prevalence of DM in Yazd province in the population over 30 years old was reported from 13.8% in 1998 to 16.3% in 2012 [13, 14]. Recent studies have reported the prevalence of the DM in 40–80-years old group 24.5% [15]. However, no comprehensive, current and representative data is available for this prevalent disease in Yazd. This study was undertaken to estimate; a) the prevalence of type 2 diabetes (T2DM) and pre-diabetes in the adult population of Yazd, b) to estimate adult un-awareness of diabetes, c) to assess the quality of care of patients in controlling the disease and its complications and d) to estimate the extent that prevalence of T2DM is affected by socioeconomic factors including gender, age group, education, ethnicity and immigration, marital status, employment and health insurance.

## Methods

### Setting, study design and data collection

**Fig. 1:**
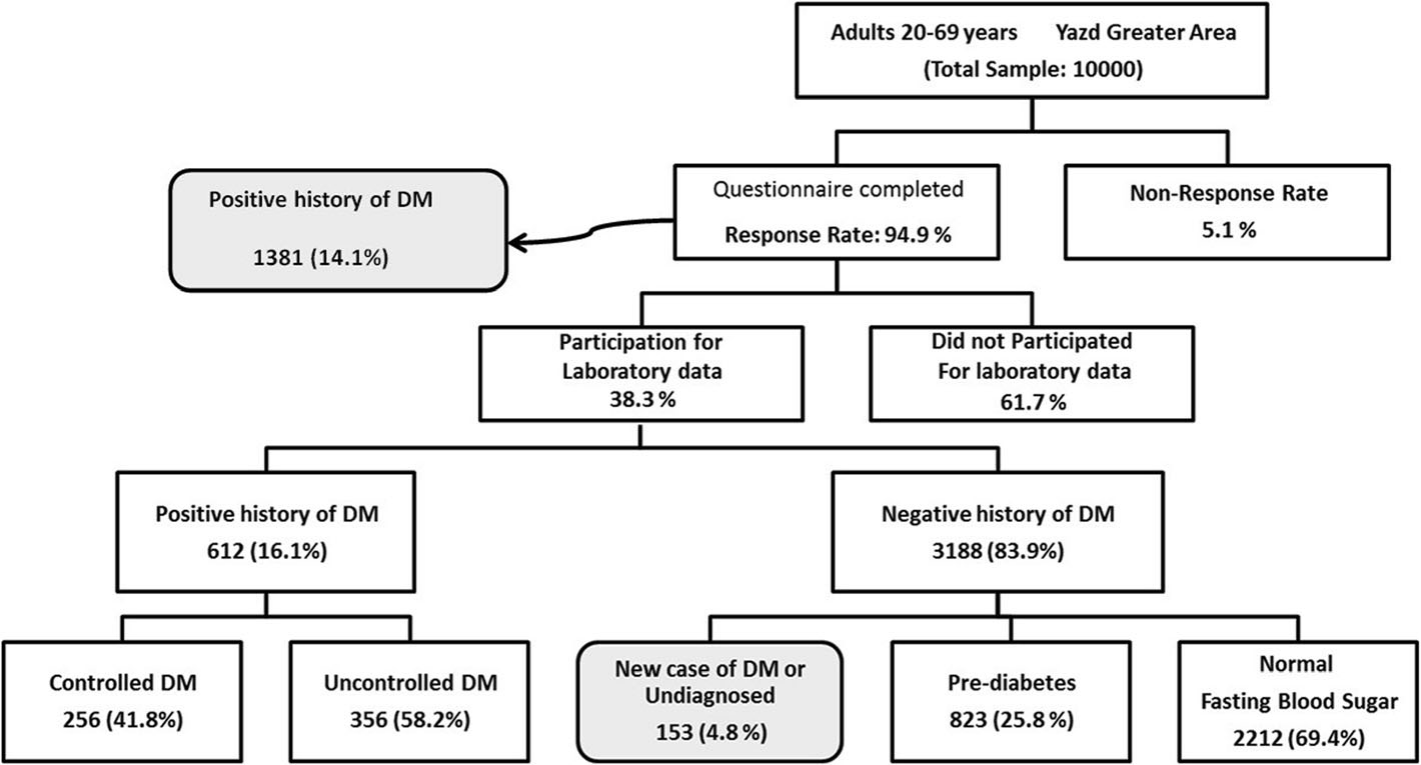
Flow diagram of participants in Yazd Health Study, who respond to questionnaires and agreed for fasting blood glucose sampling. (2014–2015)

Yazd Health Study (YaHS) is a prospective cohort study conducted to determine the prevalence of non-communicable disease and related risk factors in Yazd Greater Area. Yazd is a World Heritage City recognized by UNESCO located in the center of Iran. The sampling procedure of the YaHS study has been published elsewhere [16]. Briefly, 10,000 residents of Yazd city at the age of 20 to 69 years were selected using cluster random sampling method. At first, 200 clusters were randomly selected based on the zip code. Then, each cluster of 50 samples was divided into the following subgroups: 25 men and 25 women; five people in each age group. Each group consists of 10 people in the age group of 20–29, 30–39, 40–49, 50–59 and 60–69 years old. Inclusion criteria were ages 20–69 years at the time of the interview and completed informed consent to participate in the study (94.9% response rate) (Fig. 1).

The interviewers went to the houses of the selected individuals and coordinate for a meeting at their home to complete the questionnaire. A team of experts suggested and approved questions. The face validity was guaranteed by the panel and the Cronbach’s alpha of the questionnaire was 0.89% at the pilot stage. That who was guests and residing elsewhere was excluded from the study. Then, they were invited to go to the laboratory to perform a blood test. To assess the potential impact of individual-level socioeconomic on diabetes, self-reported information on education level, job status, health insurance, marital status, migration, and religion were recorded. Upon completion of the interview, an invitation card was sent to each participant to attend the laboratory before 9 am and after 10 h of fasting. In the laboratory, five ml fresh blood was taken from each participant; collected in an oxalate tube and centrifuged at standard time for biochemistry tests using calibrated instruments and biochemistry kits. All measurements were performed on a standard laboratory protocol using Pars Azmoon kits and Ciba Corning (Ciba Corp. Switzerland) auto-analyzer. Based on the study protocol, the team repeatedly reviews and measures every five years to determine longitudinal information on risk factors and health changes.*estimates of prevalence were reported according to baseline data (recruitment phase) here.* 3810 interviewees agreed to participate in laboratory sampling (about 40% response rate). Baseline Characteristics of the laboratory data group and those, who had no lab data was compared in Table 1.

**Table 1:** Baseline Characteristics of the laboratory data Group and those, who had no lab data

Variable	Laboratory Exam		Total	*P* Value
	Participant	Non-participant		
Total	3810 (38.4%)	6100(61.6%)		
Gender				
Male	1766 (46.4%)	3155 (51.7%)	4921	< 0.0001
Female	2044 (53.6%)	2945 (48.3%)	4989	
Age group				
20–29	566 (14.9%)	1397 (22.9%)	1963	< 0.0001
30–39	691 (18.2%)	1334 (21.8%)	2025	
40–49	853 (22.4%)	1196 (19.6%)	2049	
50–59	863 (22.7%)(	1106 (18.1%)	1969	
60–69	834 (21.9%)	1073 (17.6%)	1907	
Marital status				
Married	3321 (87.2%)	5109 (83.6%)	8430	< 0.0001
Single	295 (7.7%)	759 (12.4%)	1054	
Widowed/Divorced	192 (5.0%)	243 (4.0%)	435	
Insurance				
Insured	3585 (95.4%)	5676 (93.9%)	9261	0.001
Not insured	171 (4.6%)	366 (6.1%)	537	
Job-status				
Employed	1379 (36.6%)	2547 (42.3%)	3926	< 0.0001
Unemployed	1585 (42.1%)	2240 (37.2%)	3825	
Housewife	802 (21.3%)	1237 (20.5%)	2039	
Education				
Primary school and less	1118 (29.5%)	1469 (24.2%)	2587	< 0.0001
High school	1136 (30.3%)	1666 (27.4%)	2802	
Diploma& Graduate diploma	1027 (27.1%)	1905 (31.3%)	2932	
BSc	431 (11.4%)	860 (14.1%)	1291	
MSc. and Doctorate	73 (1.9%)	181 (3.0%)	254	

### Diagnosis of diabetes

The following criteria were used to consider a person diabetics and the prevalence *was calculated accordingly.* History of DM was recorded by practitioner diagnosis over a lifetime according to patients’ interviews (self-reported)*. DM was defined* as *fasting plasma glucose (FPG)* ≥ 126 mg/dl (7.0 mmol/L) by American Diabetes Association (ADA). Undiagnosed diabetes was defined as not having self-reported diabetes but having *a fasting plasma glucose (FPG)* ≥ 126 mg/dl in the blood test. Control of DM and pre-diabetes were defined as an FPG lower than 126 mg/dl and between 100 and 125.9 mg/dl (5.6–6.9 mmol/L), respectively [17–19].

### Statistical analysis

Yazd population in 2011 were used for direct sex and age-standardization. Categorical variables were presented as frequencies and percentages and the prevalence of DM control was reported as proportions. Chi-square test was used for categorical variables to analyze the differences in demographics across the groups. Multivariate logistic regression models were applied to determine the predictors of diabetes (diagnosed, undiagnosed and controlled) and pre-diabetes. Adjusted odds ratios were reported. To neutralize the effect of non-response bias, we weighted the data of the participants, who agreed for blood tests, in the analysis. Weighting was done for gender and age groups, weights were calculated by dividing the population percentage by the subsample percentage. All statistical analyses were performed using SPSS version 16.0 software. A *p*-value less than 0.05 was considered statistically significant.

## Results

Of the 9965 individuals recruited, 1378 reported having DM, a crude prevalence of 14.1% (95% CI = 13.4–14.7) which is more common in women than men (15.6% vs. 12.4%). The prevalence of diabetes increases with age. (33.8% at 60–69 years, compared to 1.3% at 20–29 years). The estimation of age-standardized prevalence of DM was calculated by sex. The standardized prevalence of diabetes in the study population (20–69) was estimated at 8% (8.9% in women & 7.0% in men), which increases with age and reaches 18% in age 40–69 yeras (20.4% in men and 16% in women). Figure 1 presents the flowchart of the study and the rate of participation. It shows a summary of the most important results.

Stratified by migration status, the prevalence of DM was 14.9% (95% CI = 13.9–15.5) in Yazd local people and 8.6% (95% CI = 7.0–10.1) in those migrated from other provinces. A difference was found between the prevalence of diabetes in different education groups (*P* < 0.001). The illiterate/elementary adults had the highest history diabetes(26.4%), and those with university education reported the lowest prevalence (4.7%). The prevalence of diabetes was higher in individuals who had health insurance (14.4%) compared to uninsured (7.2%). Table 2 shows the prevalence of diabetes according to socio-economic determinants. Self-reported DM in Zoroastrians, a religious minority, was 11.2% (95% CI = 7.0–15.2) which was not significantly different from the majority Muslim population.

**Table 2:** Socioeconomic factors associated with self-reported diabetes mellitus in Yazd greater area. 2014–2015

	A positive history of diabetes mellitus		*p*-value
	Num.	Percent (95% Confidence Interval)	
Gender			
Men	606	12.4 (11.5–13.3)	*< 0.0001*
Women	772	15.6 (14.6–16.6)	
Total	1378	14.1 (13.4–14.7)	
Age group			
20–29	26	1.3 (0.8–1.8)	*< 0.0001*
30–39	62	3.1 (2.3–3.8)	
40–49	182	8.9 (7.7–10.2)	
50–59	472	24.1 (22.2–26.0)	
60–69	644	33.8 (31.7–35.9)	
Education			
Primary school and less	680	26.4 (24.7–28.1)	*< 0.0001*
High school	412	14.8 (13.5–16.1)	
Diploma and graduate diploma	220	7.5 (6.6–8.5)	
BSc	60	4.7 (3.5–5.8)	
MSc. and doctorate	12	4.7 (21.-7.3)	
Positive family history of diabetes mellitus			
Yes	908	24.5 (23.1–25.9)	*< 0.0001*
No	460	8.1 (7.4–8.9)	
Employment			
Employed	307	7.9 (7.0–8.7)	*< 0.0001*
Unemployed	627	16.5 (15.3–17.7)	
Housewife	424	20.9 (19.1–22.7)	
Health insurance			
Not insured	38	7.2 (4.9–9.4)	*< 0.0001*
Iran Health Insurance Organization	275	19.8 (17.7–21.9)	
Social Security Organization	919	13.3 (12.5–14.0)	
General health insurance	32	14.8 (10.0–19.6)	
Others	102	15.0 (12.3–17.7)	
Migration status			
Native	1100	14.9 (14.0–15.7)	*< 0.0001*
From within the province	143	15.3 (13.0–17.6)	
From other provinces	108	8.6 (7.0–10.1)	
From overseas	28	13.1 (8.6–17.7)	
Marriage status			
Married	1229	14.7 (13.9–15.4)	*< 0.0001*
Single	24	2.3 (1.3–3.2)	
Widowed	132	34.9 (30.1–39.7)	
Divorced	2	3.6 (0.0–8.7)	
Religion			
Muslim	1346	14.2 (13.5–14.9)	0.216
Zoroastrian	26	11.1 (7–15.2)	

Of the total population, 1.7% (8.0% of DM patients) reported having diabetes mellitus for less than one year. According to the results, it is estimated that the incidence of disease was just about 1.1% in this age group. Oral medications or insulin in 86.8% of individuals with DM were used to control the disease. Regular use of the medications has been reported in 84.1% of patients (95% CI: 80.8–86.9). Over the last year, 91% of the patients referred to a physician, 67.1% had been visited by a specialist physician in the same period. Table 3 shows the details of DM management in Yazd population.

**Table 3:** Duration of self-reported diabetes mellitus and diabetes care behaviors in Yazd by sex 2014–2015

	Gender				Total		*P*-value
	Male		Female				
	Num.	Percent (95%CI)	Num.	Percent (95% CI)	Num.	Percent (95% CI)	
Duration of diabetes mellitus (years)							
< 1	39	6.6 (4.9–8.9)	68	9.1 (7.3–11.4)	107	8.0 (6.7–9.6)	0.110
1–2	92	15.6 (12.9–18.7)	138	18.5 (15.9–21.5)	230	17.2 (15.3–19.4)	
3–4	102	17.3 (14.4–20.5)	139	18.7 (16.0–21.6)	241	18.1 (16.1–20.2)	
5–6	101	17.1 (14.3–20.4)	108	14.5 (12.2–17.7)	209	15.7 (13.8–17.7)	
= > 7	258	43.4 (39.4–47.4)	744	39.1 (35.7–42.7)	547	41.0 (38.4–43.7)	
Type of medication							
Food regimen	19	3.8 (2.5–5.9)	33	5.6 (4.0–7.8)	52	4.8 (3.7–6.3)	0.017
Traditional	7	1.4 (0.6–2.9)	22	3.8 (2.5–5.6)	29	2.7 (1.9–3.8)	
Oral drug	335	67.8 (63.6–71.8)	393	67.2 (63.3–70.9)	728	67.5 (64.6–70.2)	
Insulin	96	19.4 (16.2–23.1)	112	19.1 (16.2–22.5)	208	19.3 (17.0–21.7)	
I don’t take medication	37	7.5 (5.5–10.1)	25	4.3 (2.9–6.2)	62	5.7 (4.5–7.3)	
Do you take medication regularly for diabetes?							
Yes	456	84.1 (80.8–86.9)	561	84.0 (81.0–86.6)	1017	84.0 (81.9–86.0)	0.943
No	86	15.9 (13.0–19.2)	107	16.0 (13.4–18.9)	193	16.0 (14.0–18.1)	
When was the last time you visited your doctor?							
3–6 months	415	76.1 (72.2–79.5)	525	76.3 (72.9–79.3)	940	76.2 (73.8–78.5)	0.789
7–12 months	78	14.3 (11.6–17.5)	105	15.3 (12.8–18.1)	183	14.8 (13.0–16.9)	
2–3 years	32	5.9 (4.2–8.2)	38	5.5 (4.0–7.5)	70	5.7 (4.5–7.1)	
4–10 years	8	1.5 (0.7–2.9)	11	1.6 (0.9–2.8)	19	1.5 (0.9–2.4)	
= > 10 years	12	2.2 (1.3–3.8)	9	1.3 (0.7–2.5)	21	1.7 (0.1–2.6)	
Which specialist doctor did you visit?							
General physician	183	31.8 (28.1–35.7)	244	33.7 (30.3–37.2)	427	32.8 (30.3–35.5)	0.604
Internal medicine	273	47.5 (43.4–51.6)	324	44.7 (41.1–48.3)	597	45.9 (43.2–48.6)	
Endocrinologist	119	20.7 (17.6–24.2)	157	21.7 (18.8–24.8)	276	21.2 (19.1–23.5)	

The results of the FPG of 3810 people (approximately 40% of the participants) show that 4.0% (95% CI: 3.4–4.7) of people, were not aware of DM which was increased with age (*p*-value < 0.0001). Table 4 shows frequency of DM, pre-diabetes, undiagnosed DM and uncontrolled DM in Yazd adult population, who participated in the study and gave blood for tests. All prevalence estimates were weighted on the basis of the age and sex variables, that are under- or overrepresented in the subsample.

**Table 4:** Prevalence of diabetes mellitus, Pre-diabetes, undiagnosed & uncontrolled DM in Yazd greater area (2014–2015)

Diabetes status	participants, who agreed for the blood sample		Adjusted weighted estimation ^a^
	Unweight	Weighted	
Pre-diabetes	21.7% (20.4–23.1)	20.7% (19.5–22.1)	17.7% (16.9–18.4)
Total DM ^b^	20.1% (18.8–21.4)	18.1% (16.9–19.3)	10.9% (10.3–11.5)
Self- reported DM	16.1% (14.9–17.3)	14.4% (13.4–15.6)	8.3% (7.8–8.8)
Undiagnosed DM	4.0% (3.4–4.7)	3.7% (3.1–4.3)	2.6% (2.3–2.9)
Uncontrolled DM	58.2% (54.2–62.0)	58.1% (54.0–62.2)	47.1% (44.2–50.1)

^a^Age and sex standardized by census data

^b^Sum of self-reported diabetes and undiagnosed

Undiagnosed diabetes was more common in men than in women (4.0% vs. 3.7%). Blood glucose was not controlled in 58.3% (95% CI = 54.2–62.1) of individuals with DM, which is not statistically significant between different age-groups and across sexes (p-value > 0.05). Prevalence of pre-diabetes was 25.8% (95% CI: 24.3–27.3) in adults. The logistic regression analysis showed that DM was higher among the women (OR: 1.4, 95% CI: (1.1–1.7)), the eldest age group (OR: 25.0). being male, younger and educated were protective factors of DM but unemployment and widow/divorced adult were high risks for it.(*p* > 0.05). In this model, there was no significant relationship between sex, education, marital status and health insurance with undiagnosed or control of DM in patients. However, higher education is a protective factor for pre-diabetes and diabetes (Table 5).

**Table 5:** Socioeconomic factors related with prevalence of diabetes, pre-diabetes, undiagnosed and uncontrolled diabetes mellitus in Yazd

	Prevalence of DM ^a^	Prevalence of DM ^b^	Undiagnosed DM ^b^	Uncontrolled DM ^b^	Pre-diabetes ^b^
	OR (95%CI)	OR (95%CI)	OR (95%CI)	OR (95%CI)	OR (95%CI)
Gender					
Male	Ref.	Ref.	Ref.	Ref.	Ref.
Female	1.4 (1.1–1.7)	1.2 (0.9–1.6)	0.7 (0.4–1.3)	1.1 (0.6–1.8)	1.2 (0.9–1.5)
Age groups					
20–29	Ref.	Ref.	Ref.	Ref.	Ref.
30–39	2.2 (1.3–3.6)	2.4 (1.0–5.8)	0.8 (0.3–2.3)	1.8 (0.2–13.3)	1.3 (0.9–1.9)
40–49	5.6 (3.5–9.0)	6.4 (2.8–14.7)	2.1 (0.8–5.5)	2.0 (0.3–13.3)	2.8 (2.0–4.1)
50–59	17.3 (11.0–27.3)	18.7 (8.3–42.2)	3.7 (1.4–9.5)	2.0 (0.3–12.8)	4.3 (3.0–6.2)
60–69	25.0 (15.8–39.7)	25.3 (11.1–57.4)	4.8 (1.8–12.5)	2.1 (0.3–13.1)	4.39 (3.0–6.5)
Education					
Primary school & less	Ref.	Ref.	Ref.	Ref.	Ref.
High school	0.9 (0.8–1.1)	0.9 (0.7–1.1)	1.0 (0.7–1.5)	0.8 (0.5–1.2)	0.8 (0.6–1.0)
Diploma &Graduate Diploma	0.7 (0.5–0.8)	0.8 (0.6–1.0)	0.5 (0.3–0.9)	1.0 (0.6–1.6)	0.9 (0.7–1.2)
BSc,MSc.Doctorate	0.6 (0.4–0.7)	0.5 (0.3–0.8)	0.5 (0.2–1.1)	0.5 (0.2–1.3)	0.6 (0.5–0.9)
Health insurance					
No	Ref.	Ref.	Ref.	Ref.	Ref.
Yes	1.4 (0.9–2.0)	0.8 (0.5–1.3)	1.7 (0.5–5.5)	0.9 (0.3–2.2)	1.6 (1.0–2.7)
Employment					
Employed	Ref.	Ref.	Ref.	Ref.	Ref.
Housewife	1.0 (0.8–1.3)	1.0 (0.7–1.4)	1.0 (0.5–1.9)	0.7 (0.4–1.3)	0.9 (0.7–1.2)
Unemployed	1.4 (1.2–1.7)	1.5 (1.1–1.9)	0.80 (0.5–1.3)	0.9 (0.5–1.4)	0.7 (0.6–0.9)
Marriage status					
Married	Ref.	Ref.	Ref.	Ref.	Ref.
Single	0.8 (0.5–1.4)	0.8 (0.3–1.7)	0.4 (0.1–1.9)	0.3 (0.1–1.9)	1.2 (0.8–1.8)
Widow/divorced	1.3 (1.0–1.6)	1.3 (0.9–1.8)	1.3 (0.6–2.5)	1.4 (0.8–2.5)	1.03 (0.7–1.5)

^a^Total sample size: 9975

^b^Subsample size, people who participated in the blood test**:** 3810

## Discussion

The present study is a descriptive analysis of diabetes status in Yazd Greater Area which addressed the frequency of DM and pre-diabetes across different age-groups, socioeconomic status, type of treatments received and awareness of the disease. According to age group distribution, Yazd has a young population structure (mean age 28.9 years), the age-standardized prevalence of diabetes estimated lower than the crude self-reported prevalence in Yazd Health Study (8% vs. 14.1%). It is expected, with an increase in the elderly population,DM prevalence increases in the future.

Our finding showed that based on FPG, 17.2% (95% CI = 16.4–18.0) of people older than 30-year-old have DM, more in women than men. Afkhami et al. in 1998 showed that 14.5% of people over 30 in Yazd province have DM [13] and in 2013, Lotfi et al., with a similar method, reported that 16% of yazd adult people have diabetes [14]. Currently, obesity in Iran is more prevalent in women than men [20] and Ghadiri et al. [21] showed that in Yazd, obesity is more prevalent in women than men. This may explain the cause of higher prevalence of T2DM among women.

The prevalence of DM has grown since 30 years ago in Iran as well as other parts of the Middle-East [22]. The lower prevalence of DM in the current study in comparison with previous studies may be due to different methods of sampling.

Zoroastrians are a religion minority in Yazd, our study showed that T2DM prevalence in this group of people is not significantly different from Muslim majority residents. Khalilzade et al. [23] in 2015 determined the prevalence of metabolic diseases in Zoroastrians and assessed DM prevalence based on FPG and Glucose Tolerance Test (GTT). They reported that total T2DM prevalence including diagnosed and undiagnosed is 26.1% among the population of older than 30 years old.

This study showed the inverse relationship between educational level and DM prevalence which is in line with other studies [24]. A low educational level can lead to harmful nutritional behaviors, obesity, lower physical activity and higher psychological stresses [25–27], all of them attributed to DM. Dray-Spira et al. reported that all-cause mortality rates in T2DM patients with lower educational levels is 28% higher than patients with higher educational level [28].

The proportion of people with T2DM who was unaware of the disease in our study was 4.8% (95% CI = 4.1–5.5), not significantly different between male and female. In a previous study in our region [14] the prevalence of undiagnosed DM was 9.0% in total population and among Zoroastrians was 18.6% [23]. In other parts of Iran, DM awareness is different. For example in Kerman- a province located south of Yazd- the prevalence of undiagnosed DM is about 2.7%, however it is 25% in the north of Iran [29, 30]. Esteghamati et al. showed that DM unawareness decreased in Iran from 45 to 24% from 2005 to 2011 [22]. Considering our study, DM awareness in Yazd is more than other parts of Iran which can be attributed to information programs of the health systems involving in DM control including Diabetes Research Center that conducts health campaigns and screening programs across the province during the past decade. High prevalence of diabetes and experience of these interventions for good awareness in Yazd, can help health managers to implement action plans for prevention and control of diabetes between study periods.Overall, we found that only aging was associated with undiagnosed DM indicating that they have a higher level of DM unawareness. Also, pre-diabetes is more common in older people; irregular care over a longer period can increase undiagnosed DM, the prevalence of uncontrolled DM was approximately 60% among persons with diabetes. This poor control is consistent with other Middle Eastern studies [31]. Socioeconomic factors such as education, employment, and health insurance did not influence controlling the disease, different from other studies [32, 33].

Our result showed that 19.3% of DM patients in our region are on insulin and the rest of patients receive oral antidiabetic agents (OAD) or diet or both. Prospective data analysis from the registry of out-patient university-affiliated clinics (NPPCD 2016) in Iran showed that more than 36% of patients with DM are on insulin or a combination of insulin and OAD [7]. However, the difference may be secondary to case collection. Our study is a community-based analysis from patients with diabetes, while the NPPCD-2016 included patients from referral university clinics with more advanced complications and it is clear that this group of patients is, obviously not representative of all DM patients.

The strength of our study was the large representative sample size, the most important limitations of our study was that only 40% of the study participants gave blood samples despite frequent reminders. Those who gave samples were not different from the rest according to nationality, religion, and birthplace in both sexes. Health insurance and employment were not different across the two groups in women. Other socioeconomic variables (age group and education) were different in those who gave blood samples versus who does not. Non-participants. It seems that referring to the laboratory for sampling is an important factor in non-cooperation. Sampling at home or paying a fee for car agency fare will increase participation in the next round.

## Conclusion

The current study showed a high prevalence of DM in Yazd Greater Area, of every five people over 40 years, one has diabetes mellitus. The prevalence of DM is related to socio-demographic factors which requirers attention to the role of these factors in controlling the disease. Briefly, DM is more common in women, insured, low educated, housewives, and people with positive family history of the disease and increases with age. Although more than 90% of the patients were aware of their disease, their blood glucose was not controlled in half of them. Pre-diabetes and undiagnosed diabetes is higher in lower educated, older, unemployed and housewives. However, uncontrolled diabetes was not related to socioeconomic factors. In the next round, intervention is required to increase participation in the blood test and reduce self-selection. The patients need to be controlled better and their medications should be adjusted according to their FPG values. Effective strategies are needed for DM prevention and control in this population. Design and implementation of patients’ registry and active follow up programs may be helpful.

## Data Availability

The data collected by Yazd Health Study are not open access but can be shared under conditions of collaboration and endowment. Data are available from the authors upon reasonable request and with permission of principal investigator. For further information, please visit YaHS website at www.yahs.ir / yahs.ssu.ac.ir

## Abbreviations

ADA: American Diabetes Association
CI: Confidence Interval
DM: Diabetes Mellitus
EMRO: Eastern Mediterranean region
*FPG*: *fasting plasma glucose*
GTT: Glucose Tolerance Test
NPPCD: National Program for Prevention and Control of Diabetes
OAD: Oral Anti Diabetic
T2DM: Type 2 Diabetes Mellitus
WHO: World Health Organization
YaHS: Yazd Health Study
